# Pervasive versus situational childhood ADHD: latent classes and their clinical characteristics, based on parent and teacher ratings in a large longitudinal population sample

**DOI:** 10.1007/s00787-023-02308-3

**Published:** 2023-10-17

**Authors:** Matilda A. Frick, Hugo Hesser, Edmund Sonuga-Barke

**Affiliations:** 1 Department of Psychology, Stockholm University, Albanovägen 12, 106 91 Stockholm, Sweden; 2 Department of Psychology, Uppsala University, Uppsala, Sweden; 3 Department of Medical Sciences, Uppsala University, Uppsala, Sweden; 4 School of Behavioural, Social and Legal Sciences, Center for Health and Medical Psychology, Örebro University, Örebro, Sweden; 5 Department of Behavioural Sciences and Learning, Linköping University, Linköping, Sweden; 6 Department of Clinical Medicine, Aarhus University, Aarhus, Denmark; 7 Department of Child and Adolescent Psychiatry, Institute of Psychiatry, Psychology and Neuroscience, King’s College London, London, UK

**Keywords:** ALSPAC, Pervasive and situational ADHD, Latent class analysis, Longitudinal

## Abstract

Attention-deficit/hyperactivity disorder (ADHD) diagnoses require symptoms to be present in two settings. Low levels of concordance between symptoms rated at home and school raise questions regarding this approach. The aim was to examine whether there are sub-groups with context-specific expressions of ADHD symptoms (i.e., at home or school only) with clinically significant problems sufficient to support a new diagnostic formulation. We applied latent class transition analysis to parent and teacher data (*N* = 10,476) from the Avon Longitudinal Study of Parents and Children (ALSPAC), collected at ages 8, 10, and 20 years. We examined the short-term stability of emergent classes and their childhood and adult-associated risk profiles. In addition to an *Unaffected* class (~ 45%), there was a *Pervasive Combined* class with elevated inattentive and hyperactive/impulsive symptoms at both home and school (~ 11%) and three classes with situational expressions; *School Combined* (~ 9%), *Home Combined* (~ 18%), and *School Inattentive* (~ 16%). Stability ranged from 0.27 to 0.78. The *Pervasive Combined* class was most symptomatic and impaired. *School inattentive* also displayed clinical symptom levels, whereas the *School* and *Home Combined* classes displayed subclinical levels. Different profiles regarding sex, cognition, conduct problems, and substance use emerged for the three situational classes. Distinct groupings of pervasive and situational ADHD expressions are identifiable in the general population. The isolation of a stable and burdensome *Pervasive Combined* class lends support to the current diagnostic approach. However, there are indications of situational expressions of ADHD with clinical symptom levels and associated difficulties.

**Supplementary Information:**

The online version contains supplementary material available at 10.1007/s00787-023-02308-3.

## Introduction

Attention-deficit/hyperactivity disorder (ADHD) is a common and impairing neurodevelopmental condition of excessive levels of persistent inattention and/or hyperactivity/impulsivity, with associated concurrent and future difficulties such as comorbid psychopathology, poor socio-economic status, impaired cognition, and substance abuse [1]. For a diagnosis to be made, symptoms need to be manifest in at least two contexts—most often interpreted as school and home [2]. When symptoms are expressed in only one setting no diagnosis can be made. This ensures that the condition is not just an adverse reaction to difficulties in a particular setting. However, the low correlation between parent and teacher ratings of ADHD symptoms (~ *r* = 0.40) [3], regarded as proxies for home and school manifestations, suggests that many individuals display symptoms, sometimes to an extreme degree, in just one setting. Importantly such discrepancies have been found to indicate real cross-contextual incongruence regarding symptoms of ADHD and disruptive behavior, in addition to rater differences and bias [4, 5].

Based on this, new formulations of psychopathology have been proposed, that wish to move away from psychopathology as generalized traits and consider situationally-based symptoms (such as ADHD) as discrete phenomena [5]. Despite this, the question of whether distinct groups of individuals with clinically significant situational expressions of ADHD can be identified empirically in the general population has rarely been addressed. Most focus has been on whether a school-situational clinical variant exists. Murray and colleagues used growth mixture modeling in a longitudinal cohort study and found evidence of school-situational displays of ADHD symptoms, but not for a home-situational subtype [6]. Their results indicated that low self-control predicted consistently high symptoms across time and that changing symptom levels across time was associated with time-varying factors such as poor relationships with teachers. Other work found that the school-situational form shared some characteristics with pervasive ADHD in terms of elevated levels of conduct problems compared to home-situational ADHD [7]. Preliminary evidence suggests that females may be over-represented in home-situational presentations [8], which may contribute to their low rates of diagnosis. Although this prior work suggests some support for considering setting-specific ADHD presentations, it has been limited in a number of ways—especially its neglect of the inattentive symptom domain, its focus on males, its lack of power due to small samples, and its failure to thoroughly examine clinical correlates including a broad range of comorbid issues and cognition such as IQ and various aspects of attention [6, 7, 9].

### Aims and hypotheses

To inform a potential new clinical formulation that considers situational displays of ADHD symptoms, we set out to examine expressions and stability of pervasive and situational ADHD ‘types’ and their concurrent and future clinical correlates. We aimed to address the following research questions (RQs):

RQ 1. Can latent classes of individuals with pervasive and situational expressions of ADHD symptoms be identified on the basis of parent (home) and teacher (school) symptom ratings at 8 (T1) and 10 years (T2)? We hypothesized the existence of distinct pervasive and school- and home-situational classes.

RQ 2. How do these classes differ in terms of symptom profile and severity, impairment, and co-occurring symptoms of psychopathology? Based on previous findings [7], we hypothesized that pervasive classes would be the most symptomatic and impaired and that school-situational classes would be more symptomatic and impaired than home-situational classes.

RQ 3. Is class membership stable between T1 and T2? We expected latent class membership to be relatively stable (i.e., that it would be more common to remain in the same class compared to change class) over a 2-year period.

RQ 4. Can classes at T1 and/or T2 be distinguished in terms of risk factors and long-term outcomes? Based on previous findings [7], we hypothesized that pervasive and school-situational classes would have weaker cognitive functioning and display worse long-term outcomes than home-situational classes.

## Method

### Participants

All data were collected as part of the Avon Longitudinal Study of Parents and Children (ALSPAC) [10, 11]. Pregnant women resident in Avon, UK with expected delivery dates between April 1991 and December 1992 were invited to take part in the study. Of the 20,248 eligible pregnancies, 14,541 were enrolled, with 13,988 children still alive at 1 year of age. After an attempt to bolster the initial sample with eligible cases who did not join the study originally, the total sample size for analyses was 14,901 children who were alive at 1 year of age. We used data collected at ~ 8 years (T1; *n* = 6306–8361), ~ 10 years (T2; *n* = 7576–8018), and ~ 20 years (T3; *n* = 3273–4233). Individuals with IQ > 70 (assessed at T1) and data from at least one time point were included in the main analyses, rendering a final sample of *N* = 10,476 (50.9% males).

Data were transferred to the authors after data access agreements between the University of Bristol and the authors and their respective institutions. Ethical approval was obtained from the ALSPAC Law and Ethics Committee, the Local Research Ethics Committees, and the Swedish Ethical Review Authority (Dnr. 2020-04782), in line with the Helsinki Declaration of 1975. Informed consent was implied for completed tasks and returned questionnaires, following the recommendations of the ALSPAC Ethics and Law Committee at the time. Further details about the design of the study, the data dictionary, data access, and ethical approval are available from: http://www.bristol.ac.uk/alspac/researchers/our-data/.

### Measures

#### Symptoms of ADHD, impairment, and co-occurring psychopathology at T1 and T2

Parents and teachers rated the 18 ADHD symptoms (9 inattentive and 9 hyperactive/impulsive) from the Diagnostical and Statistical Manual (DSM) on a scale from 1 to 3 (1 = “no more than others”, 2 = “a little more than others”, and 3 “a lot more than others”). Cronbach’s alpha for the different ADHD symptom domains ranged from *α* = 0.91 to 0.94. They rated four items regarding the degree to which symptoms interfered with daily functioning on a scale from 1 to 4 (1 = “not at all”, 2 = “only a little”, 3 = “quite a lot”, and 4 = “a great deal”). Cronbach’s alpha for impairment ranged from *α* = 0.82 to 0.84. They rated 15 items of emotional symptoms, conduct problems, and peer problems on the Strength and Difficulties Questionnaire (SDQ) [12] on a scale from 1 to 3 (1 = “not true”, 2 = “somewhat true”, and 3 = “certainly true”). Cronbach’s alpha for the SDQ subscales ranged from *α* = 0.51 to 0.80. Higher values indicated more symptoms.

#### Socio-economic status at T1

Mothers rated their highest education level (0 = “no qualification”, 1 = “Certificate of secondary education”, 2 = “Vocational”, 3 = “O level”, 4 = “A level”, 5 = “Degree”) and reported family take-home income per week from 1 (“less than £100”) to 5 (“more than £400”).

#### Child cognition at T1

The subtest Attention Sky Search from the Test of Everyday Attention for Children (TEA-Ch) was used to assess *selective attention* [13]. The task was to circle pairs of identical spaceships as fast as possible. The final score was the average time to correct the identification of each pair (controlling for motor speed in a second control trial). Higher values indicated poorer selective attention. The TEA-Ch has shown good construct validity [13] and has been used to assess attention abilities in ADHD [14].

The subtest Attention Dual Task from the TEA-Ch was used to asses *dual attention* [13]. An auditory stimulus was presented simultaneously as the Attention Sky Search task, and the task was to correctly identify pairs of spaceships and count the auditory tones. The final score was the average time taken to identify the pairs, divided by the proportion of correctly counted auditory signals (i.e., poor counting inflated the scores). Higher values indicated poorer dual attention.

The subtest Opposite Worlds from the TEA-Ch was used to assess *attentional control* [13]. An array of the digits 1 and 2 were presented, and the task was to say the opposite for each digit as fast as possible, inhibiting the pre-potent response (i.e., say “two” when 1 was presented). The final score was the total time spent on the task (which was not completed until correct responses had been made). Higher values indicated poorer attentional control.

Participants completed a short-form alternate item Wechsler Intelligence Test for Children, 3rd edition to estimate *IQ* [15].

#### Maternal mental health at T1

Mothers completed the 10 depression items on the Edinburgh Postnatal Depression Scale (EPDS) on a scale from 1 (“most of the time”) to 4 (“never”) [16]. Scores were reversed, so that higher mean values indicated more *depression*. Cronbach’s alpha was *α* = 0.88. Mothers also rated the 20 items of the trait scale of the State-Trait Anxiety Inventory (STAI) from 1 (“does not apply”) to 4 (“certainly applies”) [17]. Higher mean values indicated more *anxiety*. Cronbach’s alpha was *α* = 0.92.

#### Long-term outcomes at T3

Participants rated 12 items derived from the Edinburgh Study of Youth Transitions and Crime, on a scale from 1 (“not at all”) to 4 (“6 or more times”) [18]. Higher mean values indicated more *conduct problems*. Cronbach’s alpha was *α* = 0.58.

Participants rated 13 items of the short Mood and Feelings Questionnaire on a scale from 1 (“true”) to 3 (“not true”) [19]. Reversed mean scores indicated more *depression*. Cronbach’s alpha was *α* = 0.92. They also rated 7 items from the Generalized Anxiety Disorder (GAD-7) Scale on a scale from 1 (“not at all”) to 4 (“nearly every day”) [20]. Higher mean values indicated more *anxiety*. Cronbach’s alpha was *α* = 0.91.

Participants rated one item regarding being currently employed, in education, or in training (1 = “no”, 2 = “yes”), which was used as the measure for *education/employment*.

Participants reported (yes/no) ever using a list of seven drugs: cannabis, cocaine, amphetamine, inhalants, sedatives, hallucinogens, and opioids. The sum (0–7) was used as the measure for *drug use*. Cronbach’s alpha was *α* = 0.74. They also rated 10 items of the Alcohol Use Disorders Identification Test (AUDIT) on a scale from 0 to 4 [21]. The sum score value indicated the level of *alcohol use*. Cronbach’s alpha was *α* = 0.79.

### Statistical analyses

**Fig. 1:**
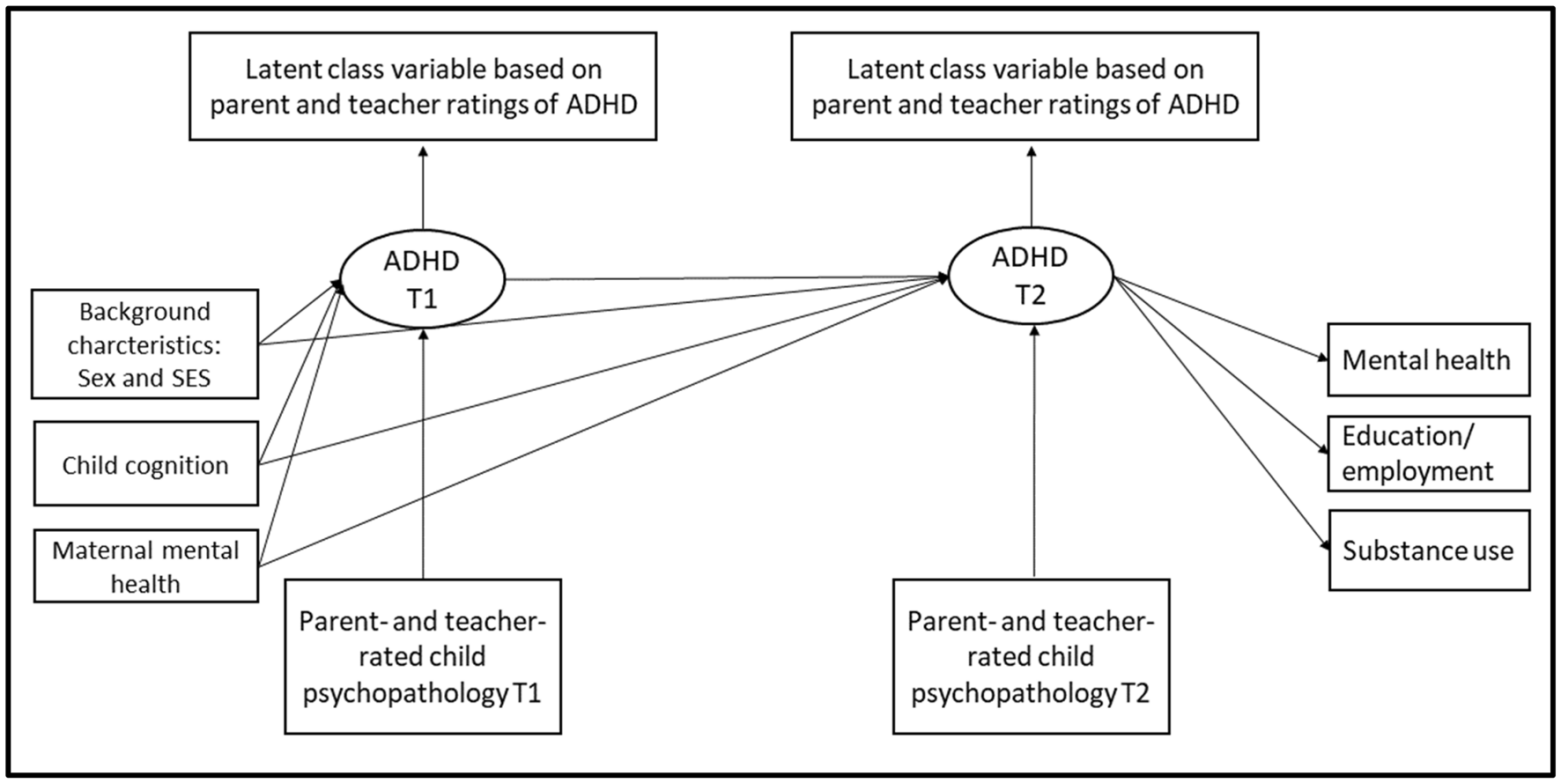
Depiction of the full model. *SES* socio-economic status, *ADHD* attention-deficit/hyperactivity disorder

The study was pre-registered on the Open Science Framework (link: https://osf.io/qvr7h/?view_only=494ca2baa06b4f019a00bca885c76704). All models were estimated with Mplus (version 8.6). To handle missing data, we used a modified joint likelihood approach, which allowed us to include participants with data from at least one data point in the analyses under the missing at random assumption [22]. See Fig. 1 for a graphical representation of the overall analytical model.

To answer RQ 1, we used latent class analysis (LCA) at T1 and T2 to evaluate subgroups within the population [23]. One to 10 classes were estimated with the expectation–maximization algorithm (500 random sets of starting values and 100 final stage optimizations). The individual ADHD symptoms as rated by parents and teachers (in total 36 items) served as categorical indicators. Each item was dichotomized, in that a rating of 1 = non-endorsement and a rating of 2 or 3 = symptom endorsed. We compared models using commonly employed fit statistics: Bayesian Information Criteria (BIC), Akaike Information Criteria (AIC), sample-adjusted BIC, and *p* value for Vuong–Lo–Mendell–Rubin likelihood ratio test (VLMR-LRT). BIC is commonly considered the most reliable indices and the VLMR-LRT has been criticized for relying on incorrect assumptions [24]. Thus, BIC was the primary fit index used to assess the relative fits of the models. If ICs do not reach minimum values, BICs can be plotted to allow for visual inspection to find an “elbow” that is the last relatively large decrease in BIC [23, 25]. We computed entropy as a measure of the quality of class separation, where values > 0.80 indicate good classification [26].

After deciding on the best-fit LCAs for each time point, we visually inspected the item probability plots and calculated impairment scores for each class to interpret and label the classes in accordance with RQ 2. DSM criteria for number of endorsed symptoms per symptom domain guided our interpretation: ≥ 6 endorsed items = clinical symptom levels and 4–5 endorsed items = subclinical symptom levels. We verbally labeled the average impairment level for each class, in accordance with the possible answers. For ease of interpretation, we visually present the number of endorsed symptoms for the obtained classes. The full-item probability plots are displayed in Figs. 3 to 6 in the Supplementary material. See also Table 2 in the Supplementary material for the depiction of the percentage in each class that displayed clinical levels of ADHD symptoms.

To explore the short-term stability of the classes (RQ 3), we conducted a latent transition analysis (LTA) using an autoregressive path between latent classes at T1 and T2 [23]. Finally, we examined associations between the latent classes at each time point and auxiliary information (RQ 2 and 4) [26]. That is, predictors and covariates were included in separate models to examine potential associations with the latent classes, whereas the effect of class membership on distal outcomes was assessed through pairwise comparisons across classes. We used the three-step specification to assure that the class solutions were not impacted by the auxiliary variables [25]. As the output for the auxiliary analyses is extensive, we present statistically significant effect sizes in abbreviated tables. To adjust for multiple testing, we used Bonferroni correction based on the 21 included auxiliary variables, which yielded a *p* value of 0.0024.

## Results

### Latent class structure

The summary of fit statistics at T1 and T2 is reported in Supplementary material Table 1. As is commonly the case, the IC fit indices did not reach minimum values. The VLMR-LRT proposed four or seven classes at T1 and one or seven classes at T2. According to the plotted BICs (see Fig. 1 in Supplementary material), the first elbow occurred at three classes at both time points, but these classes were deemed too broad, depicting low symptom levels (51.6%), medium symptom levels (32.6%) and high symptom levels (15.8%) across settings. Thus, interpretability was limited as we wished to examine situational displays of symptoms. The second elbow occurred at five classes at both time points. Examination of the item probability plots for the five-class solutions revealed that class solutions were very similar at both time points and explained the heterogeneity of symptom dispersion in a coherent way. Taken together, we decided on the five-class solution for both time points, which both had excellent entropy (0**.**855 and 0**.**865, respectively).

### Latent class characteristics, impairment, and co-occurring psychopathology

**Fig. 2:**
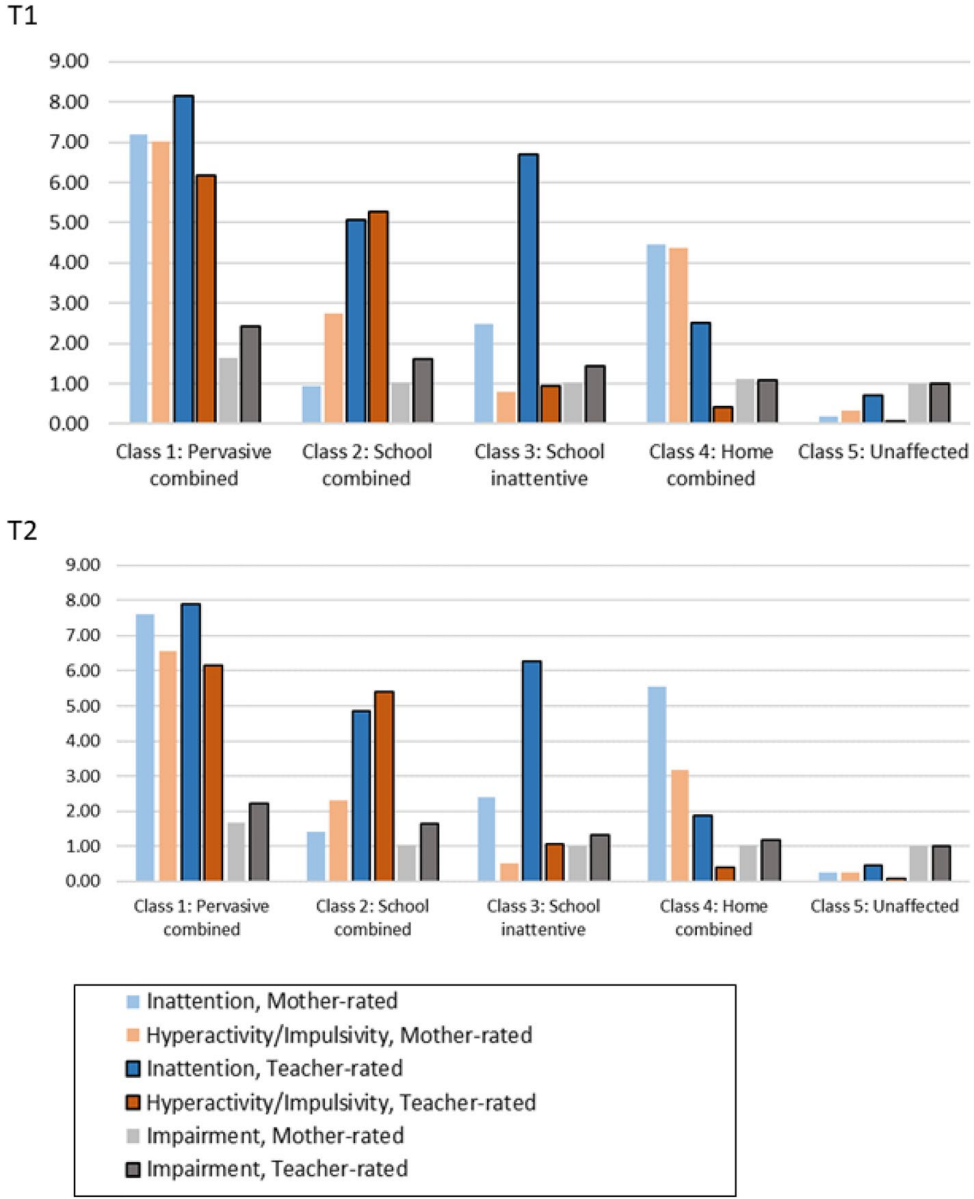
Mean values of number of endorsed ADHD symptoms and impairment for the obtained classes at T1 and T2

See Table 1 (T1) and 2 (T2) for descriptive statistics for each obtained class. See Fig. 2 for the depiction of the sum of endorsed ADHD symptoms and impairment for all classes and each setting (home versus school) at T1 and T2. The following latent classes emerged for each time point: Class 1 *Pervasive Combined* (11**.**9% at T1 and 10**.**5% at T2); Class 2 *School Combined* (9**.**5% at T1 and 9**.**2% at T2); Class 3 *School Inattentive* (17**.**1% at T1 and 15**.**3% at T2); Class 4 *Home Combined* (17**.**7% at T1 and 18**.**2% at T2); Class 5 *Unaffected* (43**.**8% at T1 and 46**.**4% at T2). *Pervasive Combined* had on average clinical levels of ADHD symptoms at home and school and the highest impairment scores (on average corresponding to between “only a little” and “quite a lot”). *School Inattentive* had on average clinical levels of inattentive symptoms in the school setting but relatively low impairment scores (on average corresponding to between “not at all” and “only a little”). *School Combined* and *Home Combined* had on average subclinical symptom levels in their respective setting and relatively low impairment scores (on average corresponding to between “not at all” and “only a little” for School combined and close to “not at all” for Home combined). *Unaffected* had very low symptom levels and low impairment scores. Most often comorbid symptoms were elevated in the setting in which the ADHD symptoms were present. Generally, *Pervasive Combined* had more conduct problems (as rated by parents and teachers) and peer problems as rated by parents than most other classes (Table 3)*.* The *Unaffected* class had lower levels of conduct problems than all other classes. In general, the *Home Combined* had elevated comorbid symptoms as rated by parents and lower comorbid symptoms as rated by teachers than the *School Inattentive* class, a pattern particularly marked at T2.

**Table 1:** The left part of the table contains descriptive statistics for all study variables for each obtained class at T1. The right part presents effect sizes (Cohen’s d) with confidence intervals for all significant class effects at T1. The adjusted p value was set to < .0024

	Descriptive statistics for all obtained classes at T1					Class comparisons at T1									
	Pervasive	Situational			Unaffected		Reference: Class 5 Unaffected				Reference: Class 1 Pervasive Combined			Reference: Class 4 Home Combined	
	Class 1 Pervasive Combined	Class 2 School Combined	Class 3 School Inattentive	Class 4 Home Combined	Class 5 Unaffected										
	*N* = 1245	*N* = 940	*N* = 1732	*N* = 1800	*N* = 4759										
						Exposure	Class 1 Perv C	Class 2 School C	Class 3 School I	Class 4 Home C	Class 2 School C	Class 3 School I	Class 4 Home C	Class 2 School C	Class 3 School I
Background characteristics															
Sex (% females)	25.4%	39.1%	44.8%	47.9%	59.2%		− 0.89 (− 0.98 to − 0.79)	− 0.52 (− 0.61 to − 0.43)	− 0.34 (− 0.42 to − 0.26)	− 0.27 (− 0.35 to − 0.20)	0.37 (0.24 to 0.49)	0.55 (0.44 to 0.65)	0.61 (0.51 to 0.72)	− 0.25 (− 0.36 to − 0.14)	
	M (SD)	M (SD)	M (SD)	M (SD)	M (SD)										
Family income	3.82 (1.20)	4.07 (1.12)	4.14 (1.09)	4.06 (1.12)	4.20 (1.05)		− 0.08 (− 0.13 to − 0.03)					0.10 (0.04 to 0.25)			
Maternal education	3.05 (1.28)	3.21 (1.23)	3.30 (1.24)	3.21 (1.28)	3.36 (1.18)										
Child cognition															
Selective attention	5.72 (2.19)	5.15 (1.64)	5.50 (1.82)	5.19 (1.80)	4.94 (1.52)				0.06 (0.03 to 0.09)						
Dual attention	16.56 (23.05)	11.46 (13.77)	12.27 (11.39)	12.65 (15.95)	10.23 (10.68)										
Attentional control	18.69 (4.70)	16.96 (3.63)	18.06 (4.29)	17.69 (4.05)	16.67 (3.46)		0.04 (0.02 to 0.05)		0.03 (0.02 to 0.05)	0.03 (0.02 to 0.04)	− 0.04 (− 0.06 to − 0.02)				
IQ	98.24 (15.20)	105.76 (15.38)	101.97 (15.31)	104.11 (15.67)	108.17 (15.09)		− 0.02 (− 0.02 to − 0.02)		− 0.02 (− 0.02 to − 0.01)	− 0.01 (− 0.01 to − 0.01)	0.02 (0.01 to 0.02)		0.01 (0.01 to 0.02)		− 0.01 (− 0.01 to − 0.01)
Maternal mental health															
Maternal depression	1.80 (0.58)	1.65 (0.52)	1.59 (0.50)	1.71 (0.52)	1.52 (0.49)							− 0.32 (− 0.51 to − 0.13)			
Maternal anxiety	2.00 (0.57)	1.83 (0.54)	1.81 (0.52)	1.92 (0.53)	1.71 (0.51)		0.32 (0.15 to 0.49)		0.24 (0.08 to 0.40)	0.36 (0.22 to 0.49)					
Child psychopathology															
Emotional symptoms (mother rated)	1.40 (0.39)	1.26 (0.31)	1.32 (0.34)	1.34 (0.36)	1.26 (0.30)										
Conduct problems (mother rated)	1.54 (0.33)	1.35 (0.28)	1.30 (0.28)	1.40 (0.29)	1.16 (0.20)		1.46 (1.25 to 1.91)	0.73 (0.47 to 1.01)	0.36 (0.13 to 0.58)	1.12 (0.93 to 1.29)	− 0.74 (− 1.01 to − 0.49)	− 1.39 (− 1.39 to − 0.89)			− 0.76 (− 1.01 to − 0.52)
Peer problems (mother rated)	1.37 (0.39)	1.21 (0.26)	1.21 (0.27)	1.25 (0.30)	1.23 (0.24)		0.59 (0.34 to 0.85)			0.41 (0.22 to 0.60)	− 0.41 (− 0.66 to − 0.17)	0.43 (− 0.66 to − 0.21)			
Emotional symptoms (teacher rated)	1.38 (0.43)	1.26 (0.38)	1.38 (0.44)	1.26 (0.38)	1.16 (0.23)		0.38 (0.19 to 0.57)		0.57 (0.42 to 0.71)						1.22 (0.87 to 1.72)
Conduct problems (teacher rated)	1.72 (0.56)	1.52 (0.52)	1.28 (0.40)	1.13 (0.31)	1.20 (0.32)		1.75 (1.51 to 1.99)	1.63 (1.37 to 2.89)	0.96 (0.72 to 1.19)			− 0.79 (− 0.95 to − 0.63)	− 1.39 (− 1.65 to − 1.17)	1.30 (1.04 to 1.55)	0.62 (0.38 to 0.86)
Peer problems (teacher rated)	1.57 (0.31)	1.51 (0.28)	1.52 (0.26)	1.49 (0.21)	1.07 (0.24)										

The right part presents effect sizes (Cohen’s *d*) with confidence intervals for all significant class effects at T1. The adjusted* p* value was set to < 0.0024

**Table 2:** The left part of the table contains descriptive statistics for all study variables for each obtained class at T2. The right part presents Cohen’s d with confidence intervals for all significant class effects at T2. The adjusted p value was set to < .0024

	Descriptive statistics for all obtained classes at T2					Class comparisons at T2									
	Pervasive	Situational			Unaffected		Reference: Class 5 unaffected				Reference: Class 1 pervasive combined			Reference: Class 4 home combined	
	Class 1 Pervasive Combined	Class 2 School Combined	Class 3 School Inattentive	Class 4 Home Combined	Class 5 Unaffected										
	*N* = 1152	*N* = 952	*N* = 1607	*N* = 2034	*N* = 5170	Exposure	Class 1 Perv C	Class 2 School C	Class 3 School I	Class 4 Home C	Class 2 School C	Class 3 School I	Class 4 Home C	Class 2 School C	Class 3 School I
Background characteristics															
Sex (% females)	25.8%	33.5%	38.3%	49.7%	60.3%		− 0.89 (− 1.01 to − 0.79)	− 0.68 (− 0.79 to − 0.58)	− 0.55 (− 0.65 to − 0.47)	− 0.24 (− 0.32 to − 0.16)		0.35 (0.22 to 0.47)	0.66 (0.54 to 0.78)	− 0.44 (− 0.56 to − 0.32)	− 0.31 (− 0.42 to − 0.21)
	M (SD)	M (SD)	M (SD)	M (SD)	M (SD)										
Family income	3.88 (1.16)	4.10 (1.13)	4.12 (1.09)	4.14 (1.09)	4.19 (1.06)										
Maternal education	3.05 (1.25)	3.20 (1.31)	3.25 (1.25)	3.31 (1.24)	3.36 (1.17)										
Child cognition															
Selective attention	5.69 (2.12)	5.11 (1.65)	5.40 (1.71)	5.33 (1.88)	4.95 (1.59)										
Dual attention	16.05 (22.19)	10.35 (11.19)	12.56 (14.33)	12.96 (15.42)	10.52 (11.72)										
Attentional control	19.03 (4.88)	16.80 (3.49)	17.89 (4.21)	17.70 (3.79)	16.75 (3.64)		0.04 (0.03 to 0.06)			0.02 (0.01 to 0.03)	− 0.05 (− 0.08 to − 0.03)				
IQ	97.91 (15.92)	107.20 (15.79)	101.66 (15.68)	103.66 (15.36)	108.17 (14.99)		− 0.02 (− 0.03 to − 0.01)		− 0.02 (− 0.02 to − 0.01)	− 0.01 (− 0.01 to − 0.01)	0.02 (0.01 to 0.03)		0.01 (0.01 to 0.02)		
Maternal mental health															
Maternal depression	1.78 (0.58)	1.57 (0.49)	1.59 (0.50)	1.70 (0.52)	1.54 (0.50)		0.32 (0.14 to 0.50)				− 0.66 (− 0.66 to − 0.16)				
Maternal anxiety	1.97 (0.57)	1.79 (0.52)	1.79 (0.52)	1.91 (0.54)	1.74 (0.52)					0.23 (0.09 to 0.37)					
Child psychopathology															
Emotional symptoms (mother rated)	1.44 (0.44)	1.25 (0.30)	1.27 (0.33)	1.39 (0.39)	1.25 (0.31)					0.33 (0.19 to 0.47)				− 0.55 (− 0.79 to − 0.31)	− 0.52 (− 0.71 to − 0.32)
Conduct problems (mother rated)	1.52 (0.39)	1.28 (0.26)	1.24 (0.26)	1.33 (0.30)	1.17 (0.22)		1.77 (1.50 to 2.03)	0.78 (0.51 to 1.07)	0.45 (0.17 to 0.73)	1.15 (0.96 to 1.34)	− 0.98 (− 1.27 to − 0.07)	− 1.33 (− 1.65 to − 1.01)	− 0.61 (− 0.86 to − 0.36)		− 0.70 (− 0.98 to − 0.42)
Peer problems (mother rated)	1.44 (0.42)	1.22 (0.31)	1.21 (0.28)	1.29 (0.34)	1.16 (0.24)		0.78 (0.53 to 1.03)			0.59 (0.40 to 0.77)	− 0.58 (− 0.86 to − 0.27)	− 0.60 (− 0.84 to − 0.36)			− 0.41 (− 0.65 to − 0.17)
Emotional symptoms (teacher rated)	1.46 (0.48)	1.25 (0.36)	1.36 (0.43)	1.25 (0.35)	1.18 (0.31)		0.71 (0.51 to 0.91)		0.71 (0.55 to 0.87)		− 0.49 (− 0.72 to − 0.26)		− 0.60 (− 0.79 to − 0.39)		0.59 (0.41 to 0.77)
Conduct problems (teacher rated)	1.81 (0.57)	1.63 (0.52)	1.29 (0.41)	1.15 (0.34)	1.06 (0.23)		1.96 (1.73 to 2.19)	1.84 (1.60 to 2.07)	0.98 (0.76 to 1.21)	0.56 (0.32 to 0.80)		− 0.98 (− 1.13 to − 0.81)	− 1.39 (− 1.65 to − 1.17)	1.27 (1.06 to 1.49)	0.42 (0.21 to 0.63)
Peer problems (teacher rated)	1.58 (0.32)	1.50 (0.26)	1.52 (0.28)	1.51 (0.24)	1.47 (0.19)										

The right part presents Cohen’s *d* with confidence intervals for all significant class effects at T2. The adjusted *p* value was set to < 0.0024

**Table 3:** Long-term outcomes

	Descriptive statistics					Pairwise comparisons									
	Pervasive	Situational			Unaffected		Comparison class: Class 5 unaffected				Comparison class: Class 1 pervasive combined			Comparison class: Class 4 home combined	
	Class 1 Pervasive Combined	Class 2 School Combined	Class 3 School Inattentive	Class 4 Home Combined	Class 5 Unaffected										
	*N* = 855	*N* = 755	*N* = 1275	*N* = 1654	*N* = 4419	Exposure	Class 1 Perv C	Class 2 School C	Class 3 School I	Class 4 Home C	Class 2 School C	Class 3 School I	Class 4 Home C	Class 2 School C	Class 3 School I
Conduct problems	1.49 (0.04)	1.04 (0.01)	1.04 (0.01)	1.02 (0.01)	1.03 (0.01)		2.85**				2.63**	2.63**	2.75**		
Depression	1.53 (0.06)	1.76 (0.04)	1.36 (0.03)	1.46 (0.04)	1.39 (0.01)			0.91**						0.71**	
Anxiety	1.86 (0.10)	2.15 (0.06)	1.52 (0.05)	1.68 (0.06)	1.58 (0.02)			0.93**				0.50*		0.72**	
Education/employed	1.89(0.03)	1.89 (0.02)	1.95 (0.01)	1.90 (0.02)	1.94 (0.01)										
Alcohol	13.53 (0.76)	8.43 (0.41)	8.80 (0.50)	7.99 (0.31)	8.48 (0.14)		0.87**				0.86**	0.79**	0.94**		
Drugs	3.00 (0.24)	1.09 (0.11)	1.48 (0.15)	1.14 (0.09)	1.00 (0.04)		1.23**				1.14**	0.88**	1.13**		

The left part of the table contains descriptive statistics (mean values and standard error) for the distal outcomes in early adulthood based on obtained classes at T2. The right part contains effect sizes (Cohen’s *d*) for significant pairwise comparisons across classes. The adjusted* p* value was set to < 0.0024. Total *N* = 8958

### Temporal stability of class membership

See Table 4 for transition probabilities across time, obtained from the LTA. As can be seen, the results indicate both stability and change. The most common pattern was for children to remain in the same class at both time points (shown in the diagonal) and the most stable class was the *Unaffected*, in which 78% of unaffected individuals at T1 were still classified as unaffected at T2. For the symptomatic classes, stability ranged from 0.27 to 0.50, with the highest stability for *Pervasive Combined*. When participants changed class, it was usually to a less severe class, such as moving from *Pervasive Combined* to *Home Combined*; or from *School Combined*, *School Inattentive*, or *Home Combined* to *Unaffected*. It was uncommon to move from *Pervasive Combined* to *Unaffected*.

**Table 4:** Latent transition probabilities from age 8 (T1) to 10 years (T2)

	T2				
T1	1	2	3	4	5
1. Pervasive combined	**0.50**	0.08	0.14	0.24	0.04
2. School combined	0.08	**0.27**	0.13	0.18	0.36
3. School inattentive	0.07	0.07	**0.34**	0.22	0.32
4. Home combined	0.09	0.11	0.17	**0.42**	0.25
5. Unaffected	0.01	0.05	0.09	0.07	**0.78**

Stability across T1 and T2 is depicted in bold on the diagonal

### Distinguishing classes

#### Risk factors

Tables 1 (T1) and 2 (T2) present effect sizes (Cohen’s *d*) with confidence intervals for factors that were statistically significantly associated with the classes at the two time points. In general, the *Unaffected* class had more females than the other classes, while *Pervasive Combined* had more males than the other classes and *Home Combined* had more females than the school-situational classes. Of the symptomatic classes, *Pervasive Combined* was the most cognitively impaired and *School Combined* was the least impaired. *Pervasive Combined* and *School Inattentive* had lower IQs than *Home Combined.* Most symptomatic classes differed from the *Unaffected* class regarding attentional control and IQ. Maternal mental health was slightly better in the *Unaffected* class than in the symptomatic classes, which in turn did not differ from one another.

#### Long-term outcomes

Table 3 presents effect sizes (Cohen’s *d*) for statistically significant pairwise comparisons between classes on long-term outcomes. *Pervasive Combined* displayed elevated levels of adult conduct problems and substance use compared to all other classes. *School Combined* displayed elevated levels of depression and anxiety compared to the *Unaffected* and *Home Combined* classes*.*

## Discussion

To inform a new diagnostic formulation of ADHD that embraces contextual variability of symptoms, the current study explored classes of individuals with pervasive and situational expressions of ADHD symptoms and mapped their prior risk factors, associated levels of impairment and comorbid symptoms, and future outcomes in a large community sample. Latent class analysis revealed one pervasive and three situational (two school-situational and one home-situational) classes. A similar class solution was obtained across a two-year period, with moderate stability in individual class membership. Four constructs emerged as distinctly differentiating factors between the symptomatic classes: sex, IQ, conduct problems, and future substance use.

In line with our hypothesis, *Pervasive Combined* was the most burdensome class and corresponds well to symptom criteria of ADHD according to the DSM-5. *Pervasive Combined* was also the most stable symptomatic class (50% remained in the class 2 years later). Very few in this class transferred to the *Unaffected* class between T1 to T2 (which was the most stable class overall, in that 78% remained unaffected). *Pervasive Combined* contained only 25% girls (significantly less than the other classes), displayed lower IQ than all other classes with the exception of the *School Inattentive*, and displayed elevated levels of substance use in adulthood. In line with previous findings [7], the *Pervasive Combined* class had the highest levels of conduct problems as rated by both parents and teachers and self-rated in early adulthood, followed by the *School Combined* class. The prevalence of 11% is higher than the standard estimate of 5–7%, which probably reflects that our estimate is based on symptom ratings of ADHD and that our definition of endorsed symptom is rather liberal as we included ratings of 2 (“a little more than others”) and 3 (“a lot more than others”). In addition, our estimate does not include the functional impairment criterion and is not assessed by clinicians.

Our results indicate preliminary evidence of situational displays of ADHD symptoms with potential clinical significance. This seemed to be the case, especially for the *School Inattentive* class, which displayed clinical symptom levels in the inattention domain and was characterized by poor attentional control, lower IQ, and elevated emotional symptoms in school. The *School Combined* and *Home Combined* classes displayed elevated yet subclinical symptom levels. *Home Combined* had significantly more girls than *School Combined* (48% versus 39%), which corroborates prior scarce findings [8]. *School Combined* was characterized by conduct problems across childhood to early adulthood, rather than by cognitive deficits. *Home Combined* was characterized by comorbid symptoms as rated by the parents, which was especially marked at T2. Taken together, we cannot confirm our hypothesis that school-situational classes are more symptomatic and impaired than the home-situational class. Instead, differential patterns of both symptoms and associated difficulties emerge.

Impairment scores for the situational classes were generally low, on average corresponding to less than “only a little”. The stability of the situational classes was also lower than for *Pervasive Combined*, and although a majority remained in the same situational class (27–42%) or transferred to another symptomatic class (36–39%), a substantial minority (25–36%) had shifted to the *Unaffected* class two years later. Interestingly, stability for the established presentations (i.e., combined, primarily inattentive, and primarily hyperactive/impulsive) is similar [27] to what we found for the situational presentations. In all, our interpretation, based on previous findings of stability, is that we found evidence for *relative stability* of class membership, although change across time is also clearly present. As such, we deem our hypothesis as partly confirmed.

Exploratory analyses showed that 34–44% of the individuals within the diverse, large situational groups displayed clinical symptom levels (i.e., fulfilled 6 or more symptoms within the specific context), whereas 99.3% in the *Pervasive Combined* class displayed clinical levels and none in *Unaffected*. Taken together, our findings indicate that the situational classes are more heterogeneous and slightly less stable than the *Unaffected* and the *Pervasive Combined* classes. We conclude that further studies that examine clinical displays of situational symptoms, including impairment, are needed before new diagnostic formulations can be proposed.

The sex distribution across classes confirms that males are more prone to display ADHD symptoms, but also suggests that girls may be equally, or almost equally prone to situational presentations, which may go undetected due to low displays of behavior problems as noted by the teachers. The inverse association between ADHD symptoms/behavior problems and IQ has been established in both clinical and typically developing samples [28, 29]. Interestingly, of the two classes marked by elevated conduct problems at school (*Pervasive Combined* and *School Combined*), only *Pervasive Combined* displayed lower IQ. This indicates that pervasive, clinical levels of ADHD, rather than situation-specific behavioral problems, drive the association between ADHD and IQ. Regarding the other comorbid symptom domains, a relatively clear pattern was that the child displayed comorbid symptoms in the setting in which s/he displayed symptoms of ADHD. Furthermore, of the assessed aspects of attention and in line with prior findings, attentional control was generally the only impaired function [14]. 

Interestingly, we did not find a class that clearly corresponds to the predominantly inattentive presentation of ADHD (that theoretically would display inattentive symptoms in both settings). However, in line with studies on this presentation, the *School Inattentive* class had a larger proportion of females and less externalizing symptoms than the combined presentation [30], indicating overlap between these classes/subtypes.

### Strengths and limitations

Strengths of the current study include a large, longitudinal community sample with a wide array of predictors, covariates, and distal outcomes. However, our method of choice provides only broad strokes of classes, including both clinical and non-clinical cases. As such, more evidence is needed before a new clinical formulation can be suggested. For instance, the results need to be replicated with other methods in clinical samples, especially given the exploratory nature of latent class analysis. Of note, to avoid inflating type-1 errors with multiple testing we used Bonferroni correction, which is a conservative method. However, despite a large sample size, we acknowledge that important effects of smaller magnitude may have gone undetected. Finally, parents and teachers were not explicitly asked to rate symptoms present in their specific context. Thus, we infer that parent ratings were mostly affected by symptoms present in the home and teacher ratings were mostly affected by symptoms present in the school.

## Conclusions

In sum, we find indications of diverse situational displays of ADHD that may contain core features of a disorder (i.e., stable symptoms across time and associated impairment). We add to prior studies by considering both ADHD-symptom domains, adding a wide variety of concurrent and future correlates in a large sample considering both sexes. We advocate further examination of these potential situational subtypes in a clinical sample.

### Supplementary Information

Below is the link to the electronic supplementary material.

Supplementary file1 (DOCX 0 KB)

## Data Availability

ALSPAC data is accessed through a system of managed open access. Please read the ALSPAC access policy (http://www.bristol.ac.uk/media-library/sites/alspac/documents/researchers/data-access/ALSPAC_Access_Policy.pdf) which describes the process of accessing the data.

## Code availability

Access to the analytic code is available on reasonable request from the corresponding author, for purposes of replicating procedures.

## References

[1] Posner J, Polanczyk GV, Sonuga-Barke E (2020). Attention-deficit hyperactivity disorder. Lancet.

[2] American Psychiatric Association (2013) Diagnostic and statistical manual of mental disorder (5th edition). American Psychiatric Publishing, Washington, DC

[3] Willcutt EG, Nigg JT, Pennington BF, Solanto MV, Rohde LA, Tannock R (2012). Validity of DSM-IV attention deficit/hyperactivity disorder symptom dimensions and subtypes. J Abnorm Psychol.

[4] De Los RA, Henry DB, Tolan PH, Wakschlag LS (2009). Linking informant discrepancies to observed variations in young children’s disruptive behavior. J Abnorm Child Psychol.

[5] Dirks MA, Reyes ADL, Briggs-Gowan M, Cella D, Wakschlag LS (2012). Annual Research Review: embracing not erasing contextual variability in children’s behaviour—theory and utility in the selection and use of methods and informants in developmental psychopathology. J Child Psychol Psychiatry.

[6] Murray AL, Ribeaud D, Eisner M, Murray G, McKenzie K (2019). Should we subtype ADHD according to the context in which symptoms occur? Criterion validity of recognising context-based ADHD presentations. Child Psychiatry Hum Dev.

[7] Mannuzza S, Klein RG, Moulton JL (2002). Young adult outcome of children with “situational” hyperactivity: a prospective, controlled follow-up study. J Abnorm Child Psychol.

[8] Rettew DC, van Oort FVA, Verhulst FC, Buitelaar JK, Ormel J, Hartman CA (2011). When parent and teacher ratings don’t agree: the tracking adolescents’ individual lives survey (TRAILS). J Child Adolesc Psychopharmacol.

[9] Ho TP, Luk ESL, Leung PWL, Taylor E, Lieh-Mak F, Bacon-Shone J (1996). Situational versus pervasive hyperactivity in a community sample. Psychol Med.

[10] Boyd A, Golding J, Macleod J, Lawlor DA, Fraser A, Henderson J (2013). Cohort profile: the ‘Children of the 90s’—the index offspring of the Avon Longitudinal Study of Parents and Children. Int J Epidemiol.

[11] Fraser A, Macdonald-Wallis C, Tilling K, Boyd A, Golding J, Davey Smith G (2013). Cohort profile: the Avon longitudinal study of parents and children: ALSPAC mothers cohort. Int J Epidemiol.

[12] Goodman R (1997). The strengths and difficulties questionnaire: a research note. J Child Psychol Psychiatry.

[13] Manly T, Anderson V, Nimmo-Smith I, Turner A, Watson P, Robertson IH (2001). The differential assessment of children’s attention: the test of everyday attention for children (TEA-Ch), normative sample and ADHD performance. J Child Psychol Psychiatry Allied Discip.

[14] Heaton SC, Reader SK, Preston AS, Fennell EB, Puyana OE, Gill N (2001). The test of everyday attention for children (TEA-Ch): patterns of performance in children with ADHD and clinical controls. Child Neuropsychol.

[15] Wechsler D (1991) Manual for the Wechsler intelligence scale for children: Third edition, vol 1991. Psychological Corp., San Antonio

[16] Murray L, Carothers AD (1990). The validation of the Edinburgh post-natal depression scale on a community sample. Br J Psychiatry.

[17] Spielberger CD, Sydeman SJ, Owen AE, Marsh BJ (1999) Measuring anxiety and anger with the State-Trait Anxiety Inventory (STAI) and the State-Trait Anger Expression Inventory (STAXI). In: The use of psychological testing for treatment planning and outcomes assessment, 2nd ed. Lawrence Erlbaum Associates Publishers, Mahwah, pp 993–1021

[18] Smith DJ, McVie S (2003). Theory and method in the Edinburgh study of youth transitions and crime. Br J Criminol.

[19] Sharp C, Goodyer IM, Croudace TJ (2006). The Short Mood and Feelings Questionnaire (SMFQ): a unidimensional item response theory and categorical data factor analysis of self-report ratings from a community sample of 7-through 11-year-old children. J Abnorm Child Psychol.

[20] Spitzer RL, Kroenke K, Williams JBW, Löwe B (2006). A brief measure for assessing generalized anxiety disorder: the GAD-7. Arch Intern Med.

[21] Saunders JB, Aasland OG, Babor TF, Fuente JRDL, Grant M (1993). Development of the Alcohol Use Disorders Identification Test (AUDIT): WHO Collaborative Project on early detection of persons with harmful alcohol consumption-II. Addiction.

[22] Sterba SK (2014). Handling missing covariates in conditional mixture models under missing at random assumptions. Multivar Behav Res.

[23] Nylund KL (2007) Latent transition analysis: modeling extensions and an application to peer vicitimization. Doctoral dissertation, University of California, Los Angeles

[24] Lubke GH, Luningham J (2017). Fitting latent variable mixture models. Behav Res Ther.

[25] Nylund-Gibson K, Grimm R, Quirk M, Furlong M (2014). A latent transition mixture model using the three-step specification. Struct Equ Model.

[26] Nylund-Gibson K, Choi AY (2018). Ten frequently asked questions about latent class analysis. Transl Issues Psychol Sci.

[27] Lahey BB, Pelham WE, Loney J, Lee SS, Willcutt E (2005). Instability of the DSM-IV subtypes of ADHD from preschool through elementary school. Arch Gen Psychiatry.

[28] Goodman R, Simonoff E, Stevenson J (1995). The impact of Child IQ, Parent IQ and Sibling IQ on child behavioural deviance scores. J Child Psychol Psychiatry.

[29] Kuntsi J, Eley TC, Taylor A, Hughes C, Asherson P, Caspi A (2004). Co-occurrence of ADHD and low IQ has genetic origins. Am J Med Genet Part B Neuropsychiatr Genet..

[30] Solanto MV (2000). The predominantly inattentive subtype of attention-deficit/hyperactivity disorder. CNS Spectr.

