# Supplementary material for: Pervasive versus situational childhood ADHD: latent classes and their clinical characteristics, based on parent and teacher ratings in a large longitudinal population sample
Source: Eur Child Adolesc Psychiatry. 2023 Oct 17;33(7):2253–66. doi: 10.1007/s00787-023-02308-3 (PMC11255028; doi:10.1007/s00787-023-02308-3)
Supplement: Supplementary file 1 — Supplementary file1 (DOCX 0 KB) [file 787_2023_2308_MOESM1_ESM.docx]

**Supplementary material**

*Table 1.* Fit statistics for latent classes based on mother- and teacher rated ADHD symptoms at 8 (T1) and 10 years (T2)

| Class | LL | BIC | AIC | SABIC | VLMR-LRT *p* | Entropy |
| --- | --- | --- | --- | --- | --- | --- |
| T1 |  |  |  |  |  |  |
| 1 | -137507·11 | 275347·464 | 275086·218 | 275233·061 |  | 1 |
| 2 | -106991·42 | 214658·586 | 214128·836 | 214426·602 | 0·000 | 0·933 |
| 3 | -100747·4 | 202513·051 | 201714·799 | 202163·487 | 0·000 | 0·893 |
| 4 | -96854·046 | 195068·847 | 194002·091 | 194601·701 | 0·000 | 0·841 |
| **5** | **-94917·638** | **191538·534** | **190203·275** | **190953·807** | **0·249** | **0·855** |
| 6 | -93221·525 | 188488·811 | 186885·049 | 187786·503 | 0·032 | 0·821 |
| 7 | -92062·566 | 186513·398 | 184641·132 | 185693·509 | 0·000 | 0·799 |
| 8 | -91199·048 | 185128·865 | 182988·096 | 184191·395 | 0·354 | 0·791 |
| 9 | -90489·015 | 184051·301 | 181642·029 | 182996·25 | 0·063 | 0·783 |
| 10 | -89948·051 | 183311·876 | 180634·102 | 182139·245 | 0·016 | 0·761 |
| T2 |  |  |  |  |  |  |
| 1 | -139582·47 | 279499·666 | 279236·942 | 279385·262 |  |  |
| 2 | -106588·92 | 213856·579 | 213323·832 | 213624·594 | 0·333 | 0·942 |
| 3 | -99920·754 | 200864·277 | 200061·508 | 200514·711 | 0·000 | 0·908 |
| 4 | -95143·103 | 191652·997 | 190580·207 | 191185·85 | 0·000 | 0·859 |
| **5** | **-93107·288** | **187925·387** | **186582·575** | **187340·659** | **0·000** | **0·865** |
| 6 | -91538·037 | 185130·909 | 183518·075 | 184428·6 | 0·000 | 0·827 |
| 7 | -90216·86 | 182832·577 | 180949·721 | 182012·687 | 0·000 | 0·799 |
| 8 | -89263·306 | 181269·491 | 179116·613 | 180332·019 | 0·162 | 0·785 |
| 9 | -88597·714 | 180282·329 | 177859·428 | 179227·276 | 0·578 | 0·784 |
| 10 | -88052·518 | 179535·959 | 176843·037 | 178363·325 | 0·230 | 0·777 |

***Note.*** N = 10,476; LL = loglikelihood; BIC = Bayesian Information Criteria; AIC = Akaike Information Criteria; SABIC = sample-adjusted BIC; VLMR-LRT = Vuong-Lo-Mendell-Rubin likelihood ratio test.

*Table 2.* Depiction of correspondence between class membership and display of clinical symptom levels at T1

|  |  |  | Clinical displays of ADHD symptoms | | |
| --- | --- | --- | --- | --- | --- |
|  | N  % (n) | No ADHD  % (n) | Pervasive  % (n) | School Only % (n) | Home only  % (n) |
| Class 1 Pervasive Combined | 359 | 0.3 (1) | 99.2 (356) | 0.3 (1) | 0.3 (1) |
| Class 2 School Combined | 337 | 33.5 (113) | 22.6 (76) | 43.6 (147) | 0.3 (1) |
| Class 3 School Inattentive | 608 | 34.2 (208) | 20.9 (127) | 44.4 (270) | 0.5 (3) |
| Class 4 Home Combined | 759 | 47.6 (361) | 19.0 (144) | 0 (0) | 33.5 (254) |
| Class 5 Unaffected | 1799 | 100 (1799) | 0 (0) | 0 (0) | 0 (0) |
| Total | 3862 | 64.3 (2482) | 18.2 (703) | 10.8 (418) | 6.7 (259) |

*Note.* Informed by DSM-5 criteria A-C, clinical display was defined by ≥ 6 symptoms in at least one domain (inattention and/or hyperactivity/impulsivity) in one setting (school or home). Pervasive was indicated by these ≥ 6 symptoms and ≥ 4 symptoms in at least one domain in the other setting. Participants with full data regarding teacher and parent ratings of ADHD symptoms at T1 were included.

*Figure 1.* Depiction of decrease in Bayesian Information Criteria (BIC) values for each added class.

*
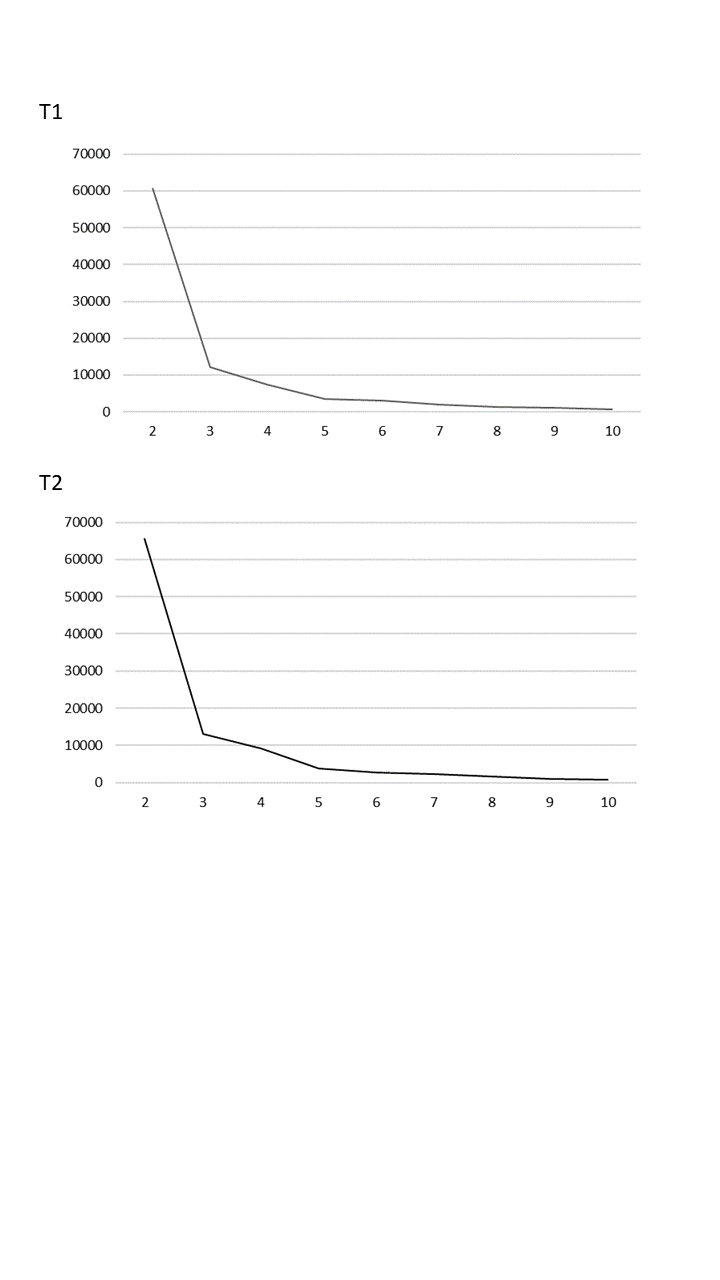
*

*Note.* Number of classes is depicted on the horizonal axis and decrease in BIC on the vertical axis.

*Figure 2.* Depiction of the rejected tree-class solution.


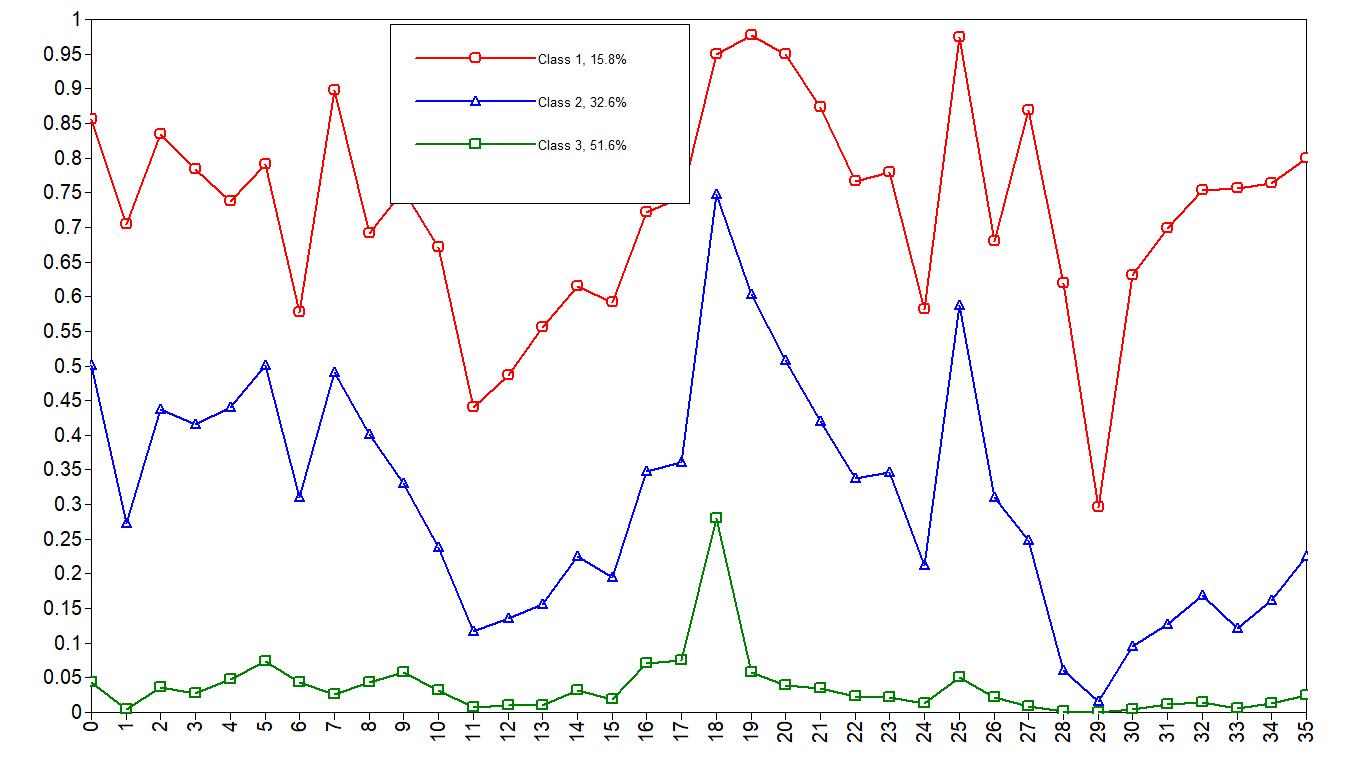

*Note.* 0-8 = parent-rated inattention; 9-17 = parent-rated hyperactivity; 18-26 = teacher-rated inattention; 27-35 = teacher-rated hyperactivity.

Figure 3. *Item probability plot for class 1 and 2 at T1*


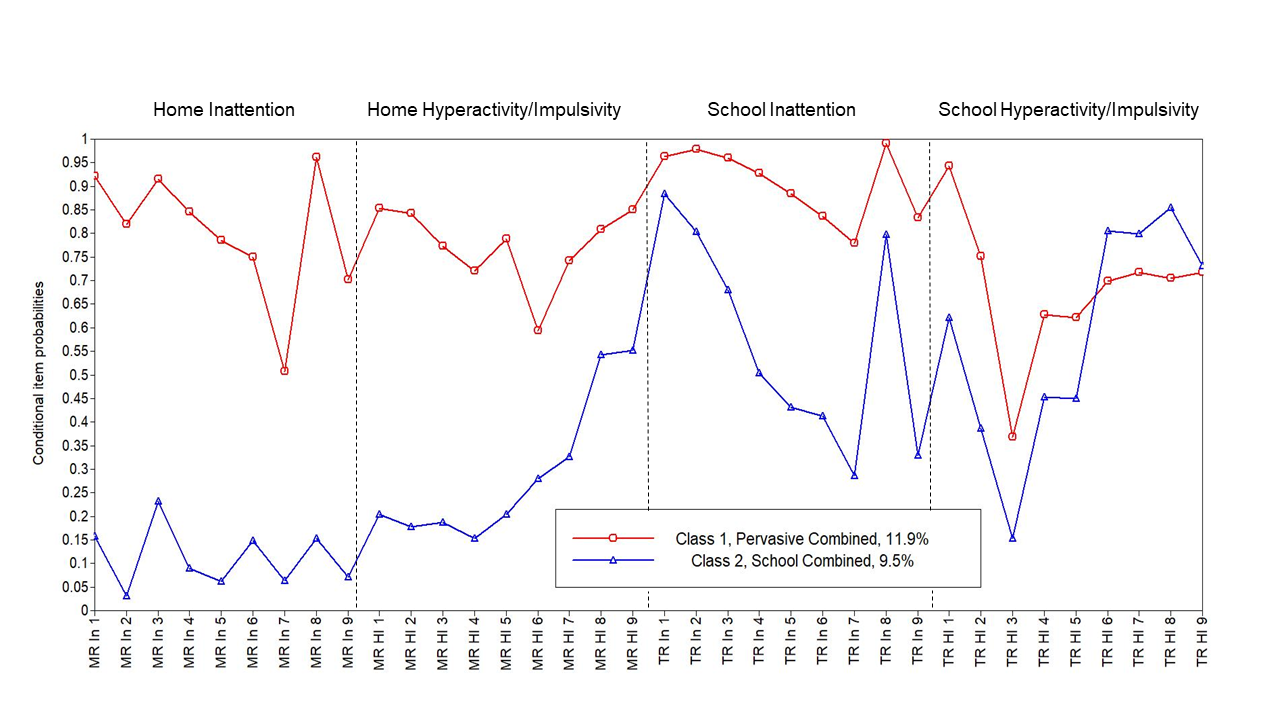


*Note.* MR = Mother rated; TR = Teacher rated; In = Inattentive symptoms; HI = Hyperactive/Impulsive symptoms.

Figure 4. *Item probability plot for class 3 to 5 at T1*


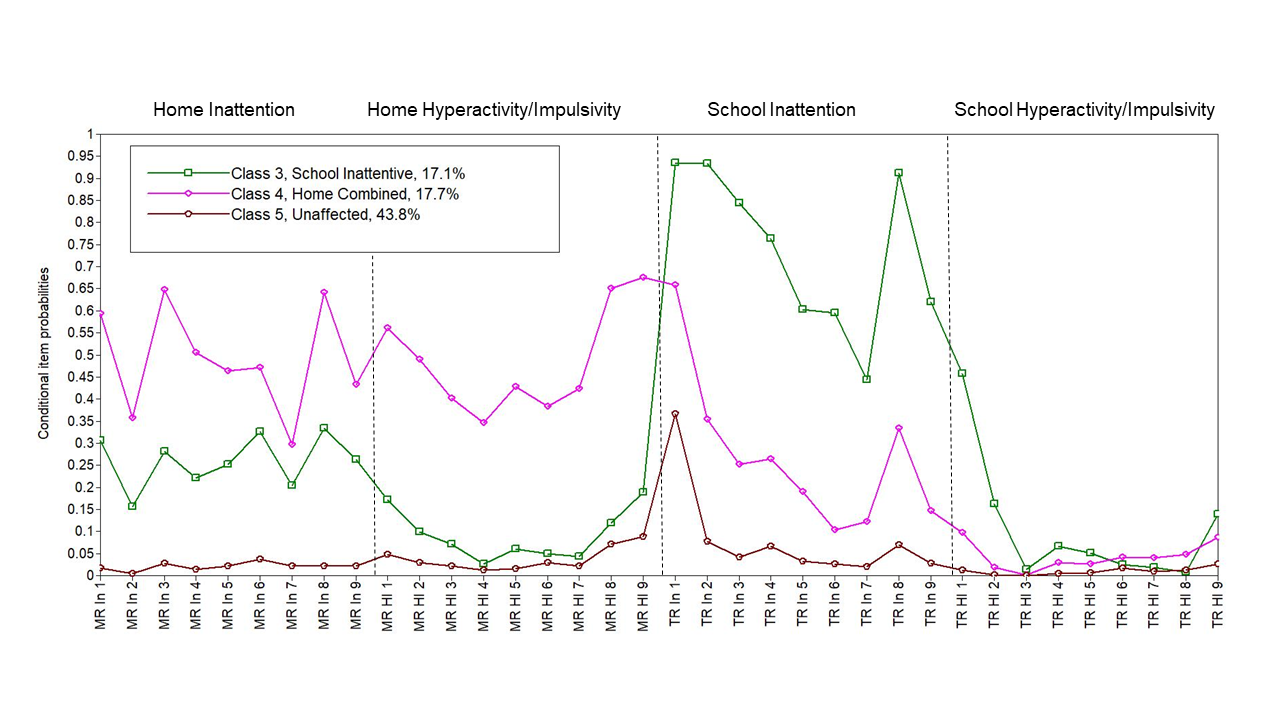


*Note.* MR = Mother rated; TR = Teacher rated; In = Inattentive symptoms; HI = Hyperactive/Impulsive symptoms.

Figure 5. *Item probability plot for class 1 and 2 at T2*


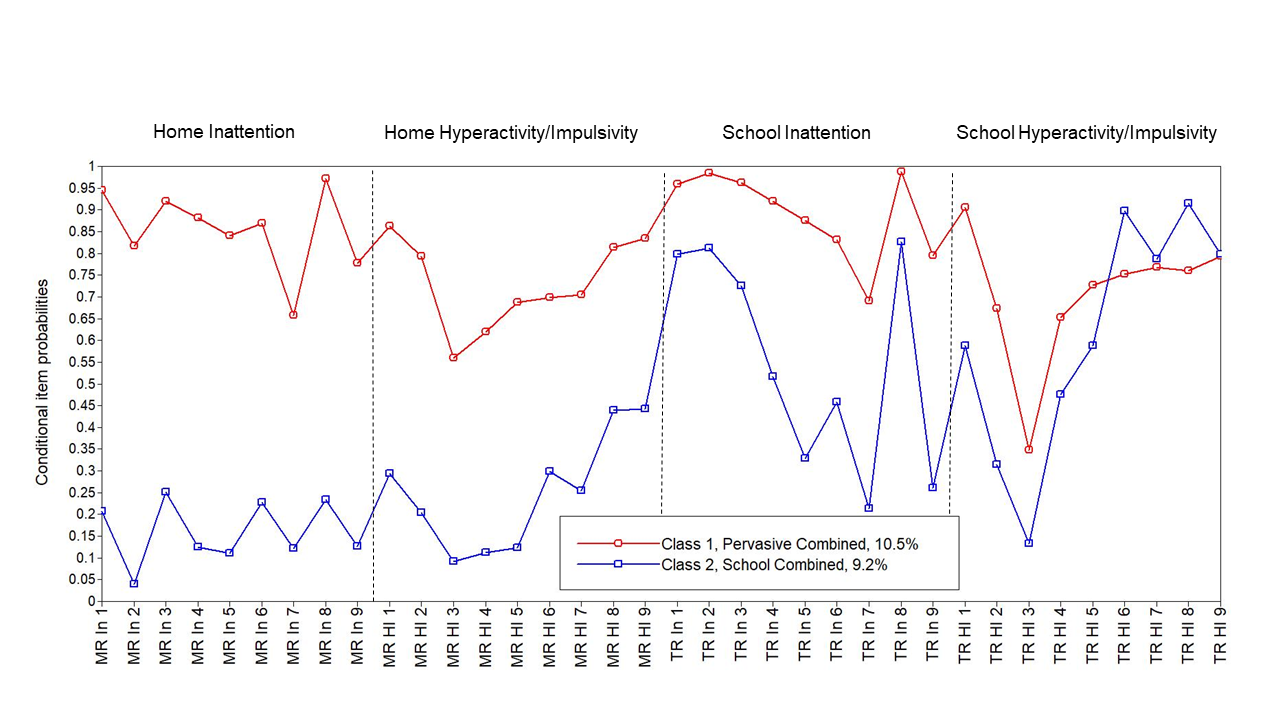


*Note.* MR = Mother rated; TR = Teacher rated; In = Inattentive symptoms; HI = Hyperactive/Impulsive symptoms.

Figure 6. *Item probability plot for class 3 to 5 at T2*


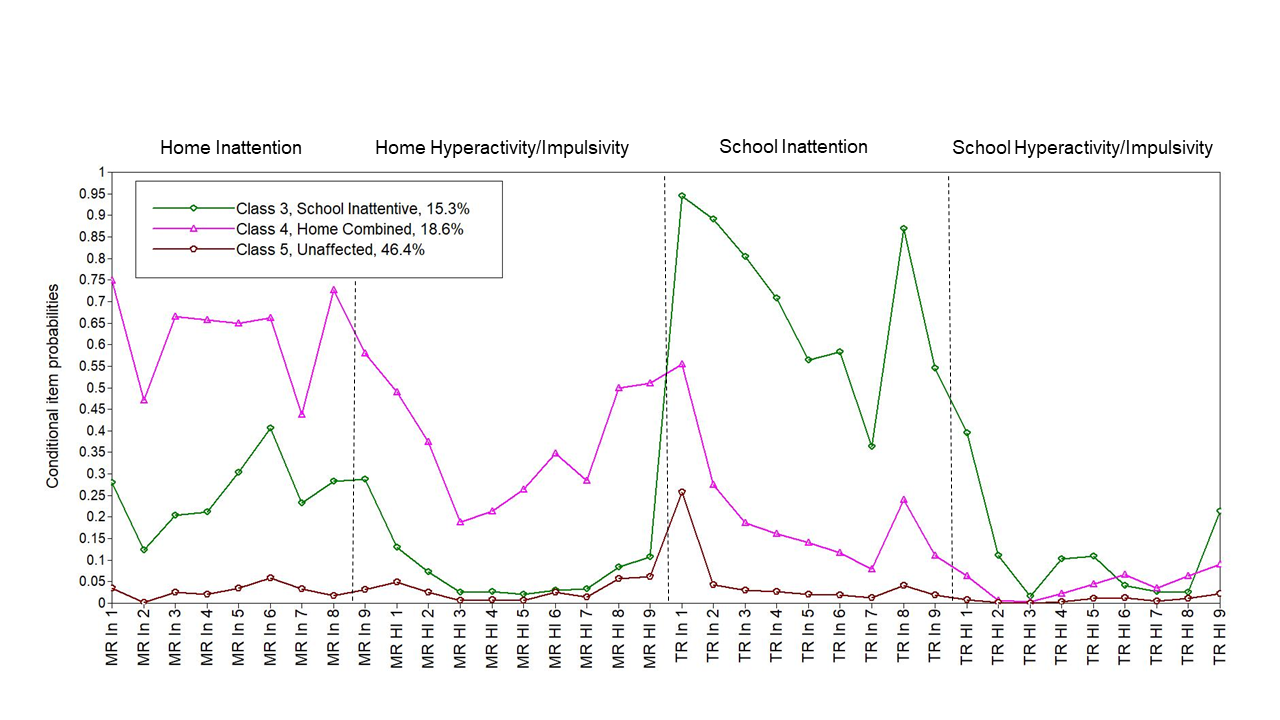


*Note.* MR = Mother rated; TR = Teacher rated; In = Inattentive symptoms; HI = Hyperactive/Impulsive symptoms.
